# A Comparative Analysis of the Advances in Stem Cell Therapy in Plastic Surgery: A Systematic Review of Current Applications and Future Directions

**DOI:** 10.7759/cureus.67067

**Published:** 2024-08-17

**Authors:** Christopher R Meretsky, Andreas Polychronis, Anthony T Schiuma

**Affiliations:** 1 Surgery, St. George's University School of Medicine, Great River, USA; 2 General Surgery, St. George's University School of Medicine, Great River, USA; 3 Orthopedic Surgery, Holy Cross Hospital, Fort Lauderdale, USA

**Keywords:** platelet-rich plasma (prp), bone marrow-derived stem cells (bmscs), adipose-derived stem cells (adscs), advances, plastic surgery, stem cell therapy

## Abstract

Stem cell (SC) therapy is revolutionizing the field of plastic surgery by harnessing the regenerative abilities of SCs derived from adipose tissue and bone marrow to boost tissue repair and enhance aesthetic outcomes. This groundbreaking method enhances results in procedures such as fat grafting, facial rejuvenation, and wound healing. As studies advance, SC therapy shows potential for more sophisticated uses in both reconstructive and cosmetic surgery. The objective of this review is to comprehensively examine the advances in SC therapy within the field of plastic surgery, highlighting its current applications and exploring future directions. The systematic review was conducted on SC therapy in plastic surgery adhering to Preferred Reporting Items for Systematic reviews and Meta-Analyses (PRISMA) guidelines and specific search criteria. This systematic review highlights these main outcomes, and SC therapy in plastic surgery enhances tissue repair and aesthetic outcomes by utilizing mesenchymal SCs such as adipose-derived SCs (ADSCs) and bone marrow-derived SCs (BMSCs), with platelet-rich plasma (PRP) providing additional support. Techniques such as scaffolds and cellular reprogramming are employed to guide SC growth, enabling tailored tissue engineering for complex regenerative procedures. This innovative approach accelerates healing, reduces scarring in reconstructive surgeries, improves skin texture, and ensures the natural integration of treated areas, ultimately yielding enhanced aesthetic results and transforming facial rejuvenation processes. SC therapy in plastic surgery holds great promise, but challenges such as protocol standardization, cost, and regulations still need to be addressed. SC therapy is leading innovative advancements in plastic surgery, offering superior outcomes and improved quality of life for patients. Interestingly, the future of plastic surgery is focused on integrating SC therapy for personalized and transformative treatments. Furthermore, interdisciplinary collaboration among bioengineers, clinicians, and regulatory bodies is essential for overcoming challenges and advancing SC research into clinical practice.

## Introduction and background

Stem cells (SCs) are undifferentiated cells capable of self-renewal and differentiating into various tissue types. This review aims to present current findings from experimental research on the use of SCs in plastic and reconstructive surgery [1].

SCs are self-renewing cells that can differentiate into multiple cell types and are classified based on their origin and differentiation potential [2]. They hold enormous potential for regenerative medicine. To develop effective SC-based treatments for various diseases, a deeper understanding of SC biology and better control over their fate is essential [3]. Additionally, addressing barriers to clinical translation, such as the potential oncogenic properties of SCs, is crucial. With renewed government support and ongoing refinement of SC methodologies, the future of SC research is promising, offering novel reconstructive options for patients and surgeons beyond traditional paradigms [4].

SCs are classified based on their differentiation potential into four categories: totipotent (or omnipotent), pluripotent, multipotent, and unipotent [5]. Omnipotent SCs, present at the earliest stages of ontogenesis, can differentiate into all types of embryonic and placental tissues. Pluripotent SCs can be harvested from the inner cell mass of blastocysts and have the ability to produce cells from all three germ layers: ectoderm, endoderm, and mesoderm. Multipotent SCs are found in almost all tissues. Initially, it was believed that they could only differentiate into cells within a single germ layer (e.g., hepatic SCs transforming solely into hepatocytes or bile ducts) [6]. However, further studies have shown that some multipotent cells possess the same potential as pluripotent SCs. Unipotent SCs have the lowest differentiation potential, generating only one cell type (e.g., epidermal SCs produce only terminal keratinized squamous epithelial cells) [7].

SCs are a crucial tool for understanding organogenesis and the body's regenerative capacity [8]. They serve as models for studying pathogenetic mechanisms and help researchers grasp the pathophysiology of various diseases [9]. Additionally, SCs offer the potential to develop biological models for testing new pharmacological agents. Their most significant potential lies in replacing damaged tissue and regenerating organs [10]. Numerous research protocols, preclinical studies, and clinical trials have been published, some of which have reported promising results for new therapeutic strategies in cell-based medicine [10]. Despite existing risks and obstacles, ongoing research and development continue to fuel optimism about the future of regenerative medicine.

The objective of this review is to comprehensively examine the advances in SC therapy within the field of plastic surgery, highlighting its current applications and exploring future directions. By detailing the various sources of SCs, such as adipose-derived and bone marrow-derived SCs, and their regenerative capabilities, the review aims to elucidate how these therapies enhance tissue repair, improve aesthetic outcomes, and address complex reconstructive challenges. Additionally, the review assesses the role of supportive techniques such as platelet-rich plasma, scaffolds, and cellular reprogramming in maximizing the efficacy of SC treatments. Furthermore, it identifies the current limitations, including regulatory, ethical, and technical challenges, and proposes potential solutions and areas for future research to advance the clinical application of SC therapy in plastic surgery.

## Review

Methods

**Figure 1:**
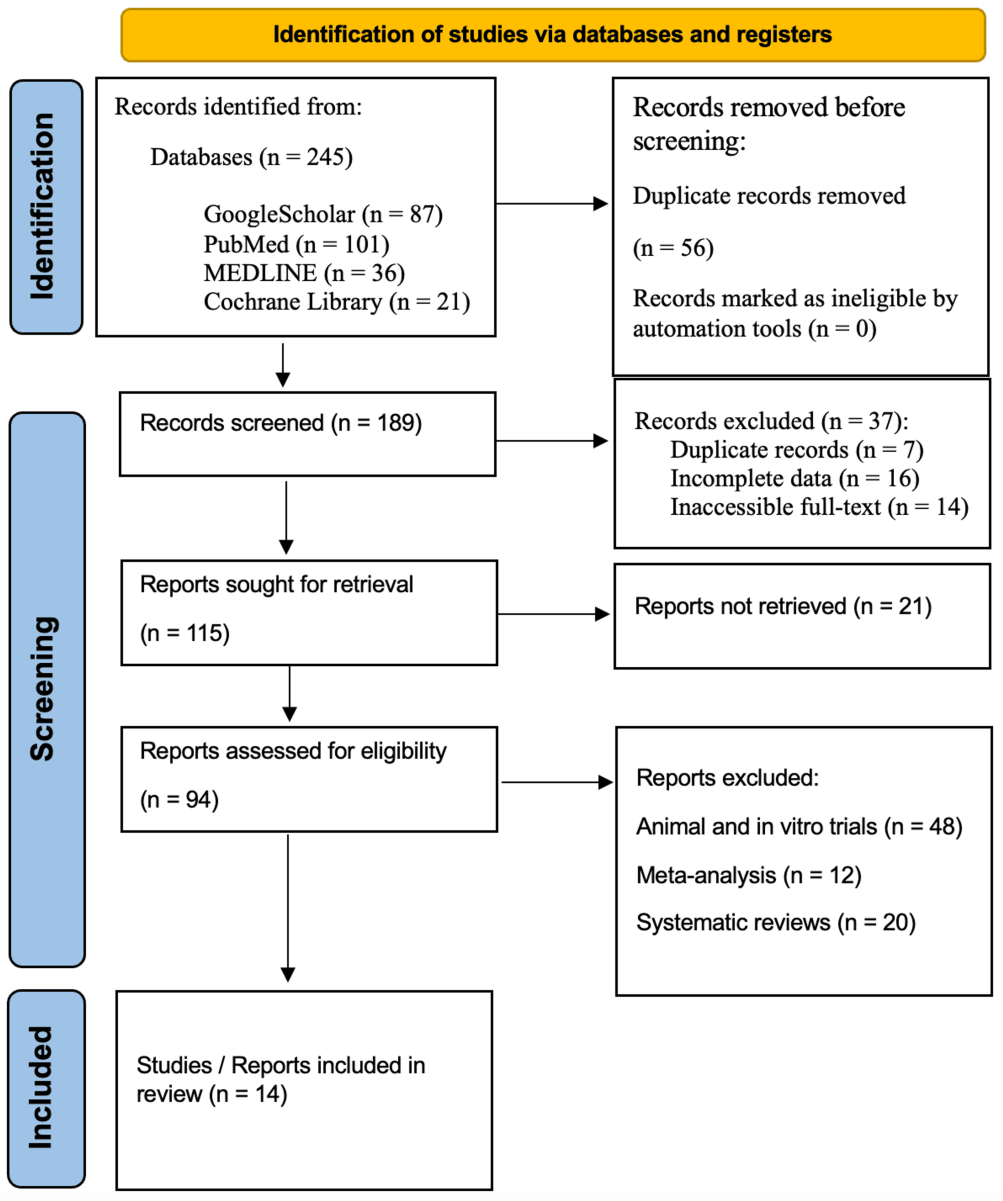
PRISMA flowchart: literature search and study selection n: number, PRISMA: Preferred Reporting Items for Systematic Reviews and Meta-Analyses Source: Ref. [11]

A systematic review was conducted in accordance with the Preferred Reporting Items for Systematic Reviews and Meta-Analyses (PRISMA) guidelines to ensure transparency and reproducibility. Comprehensive searches were performed in the Google Scholar, PubMed, Medline, and Cochrane Library databases for studies published over the past six years, from 2018 to 2024. Specific keywords used in the search included "advances in stem cells therapy," "stem cells and plastic surgery", "current applications stem cell in plastic surgery", "future directions of the application of stem cell in plastic surgery", and "challenges in the application of stem cell in plastic surgery". By adhering to the PRISMA guidelines, the review aimed to thoroughly and systematically assess the literature on these topics in Figure 1.

Inclusion Criteria

The studies considered for inclusion in this systematic review were required to meet specific criteria to ensure the relevance, quality, and comparability of the evidence presented. These criteria were established to provide a comprehensive and reliable assessment of the current state of SC therapy in plastic surgery and the clinical studies performed in humans (clinical trials).

One of the key inclusion criteria was that the studies had to involve human subjects who underwent plastic surgery procedures. This requirement ensured that the findings were directly applicable to clinical practice and patient outcomes in the field of plastic surgery. Studies involving animal models or in vitro experiments were excluded to maintain a focus on real-world clinical applications.

Another essential criterion was that the studies needed to compare different techniques of SC therapy used for plastic surgery. This could include comparisons between various SC sources (e.g., adipose-derived vs. bone marrow-derived), different methods of SC isolation and preparation, or the use of SCs in combination with other surgical techniques. By considering these factors, the review aimed to provide a comprehensive assessment of the effectiveness and safety of SC therapy in plastic surgery.

To evaluate the impact of SC therapy on patient outcomes, the included studies had to report on various factors, such as the length of hospital stay, the level of postoperative pain experienced by the patients, and the rates of postoperative infection. These outcome measures were selected to assess the potential benefits of SC therapy in terms of patient recovery, comfort, and overall surgical success.

Lastly, the studies had to be published in English to ensure that the review team could accurately interpret and analyze the findings. This criterion was necessary to maintain consistency and clarity throughout the review process. Studies published in other languages were excluded to avoid potential translation errors or misinterpretations.

Exclusion Criteria

We chose to exclude several studies from our selection criteria. Specifically, we omitted those that did not provide sufficient data on human cases of plastic surgery involving SC techniques. Additionally, we did not include meta-analyses, reviews, or editorials that did not present original findings. Research that was solely based on animal models was also disregarded. This careful selection process helped enhance the relevance and reliability of our review by concentrating exclusively on primary studies directly related to the human population of interest.

Data Extraction

After selecting the studies according to the inclusion criteria, we proceeded to extract data from them. The extracted information encompassed the study design, sample size, patient demographics, the type of sarcoma excised, the wound closure techniques employed, and the outcomes reported in each study. This comprehensive data extraction facilitated a thorough and detailed analysis of the selected studies.

Results

SC Applications in Plastic Surgery

The medical specialty of plastic surgery, also recognized as plastic, reconstructive, and aesthetic surgery, focuses on the reconstruction and reshaping of various body structures, particularly the face, following significant tissue loss. It also includes aesthetic interventions and procedures such as hand surgery, craniofacial surgery, microsurgery, and burn treatment [12].

SC therapy is an emerging field in plastic surgery that leverages the regenerative capabilities of SCs to repair and rejuvenate tissues. This innovative approach has garnered significant attention due to its potential to enhance healing processes, improve aesthetic outcomes, and address a variety of conditions that affect the skin and underlying tissues. The primary methods used in this field that we can cite are as follows: (i) mesenchymal SCs, a type of SC with regenerative potential, can be harnessed from adipose tissue (fat) through liposuction. These SCs, known as adipose-derived SCs (ADSCs), are finding increasing use in various plastic surgery procedures. In fat grafting, ADSCs are incorporated with the transferred fat to improve its survival and integration in the recipient area. Facial rejuvenation procedures can utilize ADSC injections to enhance skin texture, reduce wrinkles, and promote a more youthful appearance. Additionally, ADSCs are being explored in breast reconstruction to improve the quality and volume of the reconstructed breasts following mastectomy [13]. (ii) Bone marrow-derived SCs (BMSCs) are another type of mesenchymal SC with regenerative properties. These SCs are obtained from the bone marrow, typically the pelvic bone (iliac crest), through a two-step process. First, a bone marrow aspiration is performed to collect a sample of marrow. Then, this collected bone marrow is processed in a lab to isolate the BMSCs. Finally, the isolated BMSCs can be injected into the target area to promote tissue regeneration, aiding in wound healing and skin graft procedures [14]. (iii) Platelet-rich plasma, often abbreviated as PRP, is not a direct source of SCs, but it is a powerful tool used alongside SC therapies to boost their effectiveness. Here is how it works: First, a small amount of a patient's blood is drawn. Then, this blood undergoes a centrifugation process, which spins the blood at high speeds to separate out the PRP. Finally, this concentrated PRP is combined with the SCs before injection into the target area. PRP's role is to support cell proliferation and promote healing in the treated tissue [15]. (iv) Scaffolds are not just some random support system for SCs; they play a crucial role in guiding their growth and development. These scaffolds come in two main varieties: natural and synthetic. Natural scaffolds are derived from materials such as collagen, found naturally within the body. Synthetic scaffolds, on the other hand, are crafted from biocompatible materials, such as polylactic acid (PLA) or polyglycolic acid (PGA). The application of these scaffolds involves seeding them with SCs. These seeded scaffolds are then implanted into the target area within the body to promote tissue repair or reconstruction, for instance, in cartilage repair procedures [16]. (v) Cellular reprogramming is a powerful technique with exciting potential in regenerative medicine. This approach, known as induced pluripotent SC (iPSC) generation, involves introducing specific genes into adult cells through a process called gene editing. These reprogrammed cells, called iPSCs, then undergo a further step called differentiation, where they are guided to mature into the specific cell type needed for therapy. This ability to create customized cell populations from a patient's own cells opens doors for complex tissue engineering and regenerative procedures [17].

ADSCs

Table 1 highlights recent advancements in clinical trials (2018-2024) that have targeted adipose-derived mesenchymal SCs (AdMSC) therapy for plastic surgery. These trials showcase several innovative techniques, including the use of autologous AdMSCs. The findings from these studies indicate that the infusion of autologous AdMSCs is both safe and feasible for patients with secondary progressive multiple sclerosis (SPMS). Additionally, autologous AdMSC-based interventions show promise as a safe and effective therapy for knee osteoarthritis, with the potential to slow disease progression.

**Table 1:** Studies conducted over the last six years (2014-2018) focused on patients who have undergone plastic surgery utilizing adipose-derived mesenchymal stem cells (AdMSC) Sources: Refs. [18-21]

Reference	Method	Technique	Donor site	Subject	Nature and dose of placebo	Follow-up period	Monitored parameters	Main outcomes	Conclusion
Fernández et al. (2018) [18]	Triple-blind, placebo-controlled study	Adipose-derived mesenchymal stem cells (AdMSC)	Consenting patients underwent lipectomy, a surgical procedure to remove excess fat, and were subsequently expanded.	34 patients underwent lipectomy for AdMSCs collection	Autologous AdMSC product low-dose(1x10^6^cells/kg) or high-dose(4x1 0^6^cells/kg)	12 months	adverse events, laboratory parameters, vital signs and spirometry	One serious adverse event was observed in the treatment arms, which was a urinary infection; This event was considered unrelated to the study treatment.	The infusion of autologous AdMSCs is safe and feasible in patients with secondary progressive multiple sclerosis (SPMS).
Gentile et al. (2020) [19]	Observational case-series	Fat graft enhanced with adipose-derived stem cells (FG-e-ASCs)	Abdomen and/or flanks and/or thighs and/or inner knees	46 patients affected by breast hypoplasia	80 to 280 mL (average, 180 mL) of fat grafting was injected into each breast for a total of 360 mL (range, 160-560 mL) per patient	48 weeks	complete clinical evaluation, magnetic resonance imaging (MRI), photographic assessment, of the soft tissue, mammography (MG), and ultrasound (US)	Patients treated with FG-e-ASCs showed a 58% maintenance of contour restoration; In 67.4% of breast augmentation patients treated with FG-e-ASCs, they observed a restoration of the breast contour.	The use of fat-grafted engineered adipose-derived stem cells (FG-e-ASCs) was safe and effective in this series of cases performed.
De Celis-Ruiz et al. (2022) [20]	Randomized, double-blind, placebo-controlled, single-center, pilot clinical trial	Allogeneic Adipose Tissue–Derived Mesenchymal Stem Cells	Allogeneic adipose-derived mesenchymal stem cells (AD-MSCs), harvested from a healthy donor, were cultured in a laboratory setting.	13 patients (4 receiving AD-MSCs and 9 placebo)	10^6^cells /Kg of the patient’s weight (0.1 ml /Kg of the patient’s weight in the case of placebo solution)	24 months	Safety, including the evaluation of adverse events (AEs), assessment of neurologic and systemic complications, and monitoring for tumor development.	The total number of AEs and systemic or neurologic complications was similar between the study groups. No injection-related AEs were reported, and there was no tumor development. At the 24-month follow-up, patients in the AD-MSC group showed a non-significantly lower median National Institutes of Health Stroke Scale (NIHSS) score.	Intravenous administration of AD-MSCs within the first 2 weeks after ischemic stroke was found to be safe over a 24-month follow-up period.
Freitag et al. (2019) [21]	Randomized controlled trial	Autologous adipose-derived mesenchymal stem cell (ADMSC)	Healthy human adipose tissue	30 participants with symptomatic knee osteoarthritis	A single injection (100 × 10^6^ ADMSCs) or two injections (100 × 10^6^ ADMSCs)	6 months	Safety, pain and functional changes	No serious adverse events were reported. Both treatment groups receiving ADMSC therapy demonstrated clinically significant improvements in pain and functional outcomes at the 12-month follow-up assessment.	AdMSC therapy appears to be a safe and effective treatment option for individuals with knee osteoarthritis. This approach may have the potential to slow down or even prevent the progression of the disease.

Moreover, another technique involving fat-grafted engineered adipose-derived SCs (FG-e-ASCs) has been proven safe and effective in the cases studied. Further research demonstrates that intravenous administration of AdMSCs within the first two weeks following an ischemic stroke is safe, with a follow-up period extending up to 24 months. This method also appears to be a viable treatment option for individuals with knee osteoarthritis, potentially slowing or even preventing the disease's progression.

Overall, these advancements underscore the potential of AdMSC therapy in various medical applications, highlighting its safety and efficacy in treating conditions such as SPMS, knee osteoarthritis, and the aftermath of ischemic strokes.

BMSCs

Table 2 summarizes recent clinical trials focused on the use of BMSCs in plastic surgery. The analysis of these studies revealed several noteworthy findings. Firstly, subchondral bone marrow concentrate treatment demonstrated significant pain relief when compared to total knee arthroplasty (TKA), allowing patients with bilateral osteoarthritis to delay or potentially avoid TKA in the contralateral joint. Additionally, combining BMSC transplantation with microfracture (MFX) techniques resulted in superior postoperative healing of cartilage and subchondral bone compared to MFX alone. Moreover, bone marrow-derived allogeneic MSCs emerged as a safe and effective alternative therapy for treating peripouch fistulas in patients with Crohn's disease-like phenotype of the pouch. Furthermore, intramedullary administration of BMMSCs was found to be both safe and feasible for treating patients with acute complete spinal cord injury (SCI). Lastly, a case demonstrated that autologous human bone marrow-derived mesenchymal SCs (AHBMDMSCs) can serve as an effective alternative to autologous bone grafts and free flap transfers for treating large bone defects in the jaw.

**Table 2:** Studies conducted over the last six years (2014-2018) focused on patients who have undergone plastic surgery utilizing bone marrow mesenchymal stem cells (BMSCs) Sources: Refs. [22-26]

Reference	Method	Technique	Donor site	Subject	Nature and dose of stem cells	Follow-up period	Monitored parameters	Main outcomes	Conclusion
Hernigou et al. (2021) [22]	Randomized controlled trial	Bone-marrow mesenchymal stem cells (BMSCs)	Iliac bone marrow concentrates	140 patients, with a mean were planning to undergo staged-bilateral total knee arthroplasty (TKA) for medial osteoarthritis.	BMSCs at 7800 MSCs/mL	10 years	Osteoarthritis (OA) grade and risk of subsequent knee arthroplasty due to absence of bone marrow lesions regression.	The overall incidence of knee arthroplasty (replacement) after cell therapy was 1.19% per person-year; After accounting for confounding factors, the presence of persistent bone marrow lesions (BMLs) larger than 3 cm³ after cell therapy.	Subchondral bone marrow concentrate treatment, when compared to total knee arthroplasty (TKA), had a significant enough effect on pain relief to allow patients with bilateral osteoarthritis to postpone or potentially avoid undergoing TKA in their contralateral joint.
Hashimoto et al. (2019) [23]	Randomized controlled trial	Autologous bone marrow-derived mesenchymal stem cells (BMSCs)	Both sides of the iliac crest from the antero-superior iliac spine	11 patients with a symptomatic articular cartilage defect of the knee	BMSCs with microfracture (MFX) or MFX alone	48 weeks	Quantitative and qualitative evaluations of repair tissue	Any significant differences in the preoperative and postoperative International Knee Documentation Committee (IKDC) and Knee injury and Osteoarthritis Outcome Score (KOOS) between the cell-T group and the control group. However, 48 weeks after the surgery, the cell-T group showed a trend towards a higher KOOS quality of life (QOL) score compared to the control group.	Compared to MFX alone, the combination of BMSC transplantation and MFX resulted in better postoperative healing of the cartilage and subchondral bone.
Lightner et al. (2023) [24]	Randomized controlled trial	Allogeneic bone marrow derived mesenchymal stem cells	-	22 patients with peripouch fistulas in the setting of Crohn’s	75 × 10^6^ MSCs	18 months	Adverse and serious adverse events at post procedure	Any adverse or serious adverse events related to the MSC therapy was reported. At the 6-month follow-up, 31% of the patients in the treatment group and 20% of the patients in the control group had achieved complete clinical and radiographic healing.	Bone marrow-derived allogeneic MSCs present a secure and efficient alternative therapy method for peripouch fistulas within the context of a Crohn's-like phenotype of the pouch.
Saini et al. (2022) [25]	Randomized controlled trial	Marrow-derived mesenchymal stem cells (BM-MSCs)	Iliac crest	27 patients with acute spinal cord injury (SCI)	2 × 10^8^ cells	2 years	Respiratory issues, swelling, heightened blood pressure, irregular heartbeat, headache, fever, coldness or shivers, hives, and nerve-related pain.	3 patients dropped out, 3 patients were no longer involved in the study, and 8 patients passed away, resulting in a total of 13 patients for the final evaluation. Seven of these patients belonged to the stem cell group, while 6 were part of the control group; No negative effects linked to stem cell injections were observed based on lab and imaging results. Five patients from the control group and 3 from the stem cell group died throughout the follow-up period.	The intramedullary administration of BM-MSCs was determined to be safe and viable for treating patients with acute complete SCI.
Son et al. (2019) [26]	Case report	Autologous human bone marrow-derived mesenchymal stem cells (AHBMDMSCs)	Patient’s left anterior iliac crest	Unicystic ameloblastoma in a 14-year-old girl	AHBMDMSCs (4.8 x 10^7 ^cells/1.6 mL)	2 years	-	The case was successfully treated with mandibular regeneration using AHBMDMSCs, without the need for autologous bone grafts or free flap transfer.	This case demonstrates that AHBMDMSCs can serve as an alternative to autologous bone grafts and free flap transfers for treating large bone defects in the jaw.

Table 3 presents randomized controlled trials conducted over the past six years that investigated PRP techniques in plastic surgery. The analysis of these trials revealed several key outcomes. The application of PRP during maxillofacial surgeries significantly enhances postoperative wound healing, leading to faster tissue regeneration and reduced swelling and infiltration. Additionally, the use of PRP has been shown to significantly improve the aesthetic results of surgical procedures. Furthermore, PRP has yielded promising results in enhancing the healing of split-thickness skin grafts. The combination of adipose-derived SCs and PRP was found to accelerate refractory wound healing and reduce waiting times compared to standard dermal grafts. Moreover, combining PRP with skin flap transplants can expedite the healing process for wounds and fractures while minimizing the risk of adverse reactions during treatment.

**Table 3:** Studies conducted over the last six years (2014-2018) focused on patients who have undergone plastic surgery utilizing platelet‐rich plasma (PRP) Sources: Refs. [27-31]

Reference	Method	Technique	Donor site	Subject	Nature and dose of stem cells	Follow-up period	Monitored parameters	Main outcomes	Conclusion
Menchisheva et al. (2019) [27]	Randomized controlled trial	Platelet‐rich plasma (PRP)	Blood samples of wounds	A total of 100: 50 patients were assigned to a control group (which did not receive PPRP injections), while another 50 patients were assigned to a treatment group.	0.1 to 0.2 mL of the autologous plasma	30 days	Measurement of IL-1β, TNFα, and IL-6 cytokine levels in the wound drainage fluid	The application of PRP has shown significantly positive effects, enhancing the healing process. In the treatment group, fibroblasts, macrophages, and collagen fibers emerged and their levels increased earlier than those observed in the control group.	The application of PRP during maxillofacial surgeries significantly enhances postoperative wound healing, resulting in quicker tissue regeneration and reduced swelling and infiltration.
Менчишева et al. (2021) [28]	Randomized controlled trial	Platelet‐rich plasma (PRP)	Blood samples of wounds	100 patients who were receiving plastic and reconstructive surgery for the maxillofacial region.		30 and 90 days after the surgical procedure	The Dermatological Quality of Life Index was utilized to evaluate how treatment outcomes adversely affected different facets of the patients' lives.	The reduction in scar width was significantly less pronounced in the treatment group compared to the control group. Patients in the treatment group expressed greater satisfaction with their treatment outcomes and reported a higher quality of life.	The application of PRP significantly enhanced the aesthetic results of surgical procedures.
Tayyaba et al. (2022) [29]	Randomized controlled trial	Platelet‐rich plasma (PRP)	Blood samples of wounds	50 patients undergoing split-thickness skin grafting were enrolled due to specific clinical indications and received platelet-rich plasma treatment.	-	2 weeks	-	In the group of patients who underwent PRP therapy, there was a 100% graft uptake observed. In contrast, the control group experienced complete graft loss in 4 patients, partial graft uptake in 7 patients, and complete graft uptake in 9 patients.	This study showed encouraging outcomes for split-thickness skin grafts following the application of PRP.
Lv et al. (2024) [30]	Randomized controlled trial	Artificial dermis (AD) and autologous platelet-rich plasma	-	16 patients with refractory wounds were randomly assigned to receive either autologous PRP therapy in conjunction with artificial dermis treatment or treatment with artificial dermis alone.	-	-	Duration of wound healing, infection management, and AD vascularization, along with the number of hospital days and overall clinical outcomes.	The duration of wound healing, infection control, AD vascularization, and hospitalization after transfer was significantly reduced in the PRP + AD group compared to the AD group.	The combination of AD and PRP enhanced refractory wound healing and reduced waiting times when compared to standard dermal grafts.
Wang et al. (2021) [31]	Randomized controlled trial	Platelet-rich plasma combined with a skin flap transplant	-	72 patients with open foot fractures and associated soft tissue injuries.	-	3 weeks and 6 weeks after the treatment	The changes in wound volume before and after treatment, along with the times for wound healing, fracture healing, and any adverse prognostic reactions, were observed.	Following the treatment, wound volumes decreased in both groups, with the RG demonstrating a smaller volume compared to the CG at both 3 weeks and 6 weeks post-treatment. The average wound healing time in the RG was significantly shorter than in the CG. The average fracture healing time in the RG was also significantly lower than in the CG.	Combining PRP with a skin flap transplant can speed up the healing process for wounds and fractures while reducing the likelihood of adverse reactions during treatment.

Discussion

Over the last decade, SC-associated therapies have gained widespread use due to their self-renewal capabilities and multipotent differentiation potential. SCs have become increasingly appealing for aesthetic applications and plastic surgery, including procedures such as scar reduction, breast augmentation, facial contouring, hand rejuvenation, and anti-aging treatments [32]. Plastic surgery has a long history of using grafts from a patient's own tissues to repair injuries. Now, research suggests a single type of cell could revolutionize this field by enabling tissue regeneration [33]. Current preclinical and clinical studies on the aesthetic applications of SCs have shown promising results. ADSCs are frequently used in fat grafting, demonstrating improvements in scar appearance, anti-aging effects, and skin rejuvenation properties [34]. However, SC-based products for aesthetic medicine and plastic surgery have yet to receive FDA approval [35]. Furthermore, there is still a limited review of the efficacy and potential of SC-based therapies in the field of aesthetic and plastic surgery.

Our results reveal that recent clinical trials (2018-2024) have demonstrated the potential of AdMSC therapy in plastic surgery, highlighting the safety and feasibility of using autologous AdMSCs for conditions such as SPMS and knee osteoarthritis. Innovative techniques, including FG-e-ASCs, have shown effectiveness in treated cases. Additionally, intravenous administration of AdMSCs within two weeks of an ischemic stroke has proven safe and may offer a viable treatment option for knee osteoarthritis, potentially slowing disease progression. Overall, these advancements underscore the promising applications of AdMSC therapy in various medical contexts.

Over the past decade, numerous in vitro and in vivo studies have been conducted to develop novel strategies and techniques involving AdMSCs for use in plastic surgery. These studies have focused on optimizing the isolation, expansion, and application of AdMSCs to enhance their therapeutic potential and efficacy in various cosmetic and reconstructive procedures. Researchers have explored areas including isolation and expansion, fat grafting, wound healing and scar reduction, anti-aging and skin rejuvenation, tissue engineering, and safety and efficacy. Among these recent preclinical investigations, we can cite [36-38].

Several advances have been made in the technology that supports the use of AdMSCs in plastic surgery. In this context, in 2018, Lobascio et al. introduced a novel closed device, the MYSTEM® EVO Technology, which enables non-enzymatic tissue separation and rapid isolation of the lipoaspirate fluid from human liposuction-derived adipose tissue [39]. The authors present the case of a 77-year-old male patient who presented with a left posterior lateral perianal abscess. This abscess was associated with a fistula tract that was approximately 6 cm in length. The fistula had a posterior external opening, but an internal opening could not be identified. The experience suggests that ADSCs are a promising new sphincter-preserving treatment option for high or complex trans-sphincteric anal fistulas. Additionally, the MYSYSTEM® EVO Technology is potentially useful for this clinical application [39].

Bone Marrow Mesenchymal Stromal SCs (BMSCs)

BMSCs are plentiful in the bone marrow and are found in the body's connective tissue and organ stroma. They serve as hematopoietic and supportive cells within the bone marrow. The lack of unique surface markers makes BMSCs less likely to trigger an immune response, allowing them to evade immune surveillance easily [26].

Recent clinical trials summarized in Table 2 have explored the use of BMSCs in plastic surgery, yielding several significant findings. Notably, treatment with subchondral bone marrow concentrate provided substantial pain relief compared to TKA, enabling patients with bilateral osteoarthritis to delay or avoid TKA in the contralateral joint. Additionally, combining BMSC transplantation with microfracture (MFX) techniques resulted in improved postoperative healing of cartilage and subchondral bone compared to MFX alone. Bone marrow-derived allogeneic MSCs were identified as a safe and effective alternative for treating peripouch fistulas in patients with Crohn's disease-like phenotype. Furthermore, intramedullary administration of BMMSCs proved to be safe and feasible for patients with acute complete SCI. Lastly, a case study indicated that autologous human bone marrow-derived mesenchymal SCs (AHBMDMSCs) can effectively replace autologous bone grafts and free flap transfers for managing large bone defects in the jaw. In the same context, Nakano et al. conducted a study where they injected BMSCs three times per week into the cerebrospinal fluid (CSF) to examine the function of BMSCs for sub-acute (one to two weeks post-injury) and chronic (four weeks post-injury) SCI [26]. After transplantation, numerous axons were found extending longitudinally in the astrocyte-devoid areas, where there were initially no astrocytes or oligodendrocytes. These findings suggest that BMSCs may have the potential to promote axon regeneration and overcome the inhibitory environment created by glial scarring in SCI [26].

Linard et al. [40] investigated the effects of local autologous BMMSC injections combined with plastic surgery for treating skin necrosis in a large-animal model. Three months after irradiation overexposure to the rump, minipigs were divided into three groups: the first group underwent simple excision of the necrotic tissue, the second group received vascularized-flap surgery, and the third group received vascularized-flap surgery along with local autologous BMMSC injections. The necrotic tissue excision resulted in a pathological scar characterized by myofibroblasts, excessive collagen-1 deposits, and inadequate vascular density. Vascularized-flap surgery alone led to inadequate production of extracellular matrix (ECM) proteins (decorin and fibronectin), a low collagen-1/collagen-3 ratio, persistent inflammatory nodules, and loss of vascularization, all of which indicated continued ECM immaturity. In contrast, BMMSC therapy combined with vascularized flap surgery resulted in mature wound healing. This was evidenced by a collagen-1/collagen-3 ratio and decorin and fibronectin expression similar to that of nonirradiated skin, absence of inflammation, and vascular stability. This preclinical model demonstrated that vascularized-flap surgery successfully and sustainably remodeled irradiated skin only when combined with BMMSC therapy [40].

PRP

PRP is a concentration of autologous platelets that is abundant in biologically active components, promoting immune cell responses that reduce inflammation and enhance tissue repair and wound healing. PRP is derived from a patient's own blood for therapeutic applications and is laden with platelet derivatives that include a variety of cytokines, growth factors, chemokines, and fibrin [41]. The growth factors found in platelets are crucial for the healing process and the formation of new tissue [42].

Table 3 summarizes randomized controlled trials from the past six years on PRP in plastic surgery, revealing significant outcomes. PRP enhances postoperative wound healing in maxillofacial surgeries, improves aesthetic results, and accelerates the healing of split-thickness skin grafts. Combining PRP with adipose-derived SCs or skin flap transplants further expedites wound and fracture healing while reducing adverse reactions and waiting times compared to standard treatments.

A recent study aimed to evaluate the impact of PRP on skin repair following epidermal surgical lesions. Researchers made a clean 5 cm epidermal incision in the interscapular region of New Zealand rabbits, dividing them into two groups. Group 1 served as the control group with no treatment, while Group 2 received PRP on the surgical wound. Autologous PRP was obtained from whole blood drawn via jugular vein puncture, collected with sodium citrate anticoagulant solution, and centrifuged at 120 g for five minutes. One milliliter of the plasma phase above the buffy coat was used. Biopsies from the injured tissue were collected on days 3, 7, 14, and 21 post-surgery and subjected to hematoxylin-eosin and Masson’s trichrome staining. The study concluded that the platelet separation method effectively yields plasma with a higher concentration of platelets and fewer leukocytes. Additionally, the results indicate that therapeutic PRP administration on surgically induced skin injuries positively affects the histological features of tissue healing compared to the control group [43].

Induced Pluripotent SCs (iPSCs)

iPSCs have been utilized in clinical regenerative cell therapies for conditions such as retinal macular degeneration, spinal cord injuries, and heart failure [44-46]. However, the application of iPSCs in autologous settings is limited by the significant time and cost associated with producing individualized iPSCs. To address this challenge, a banking system for iPSCs has been proposed, which involves collecting human leukocyte antigen (HLA)-typed cells from cord blood banks, marrow donor registries, and HLA-matched platelet donor registries [47,48]. Recently, tissue transplantation derived from iPSCs through this allogeneic banking approach has been successfully conducted [49].

Recently, a system for banking iPSCs to collect HLA haplotype homozygous (homo) cells for iPSC transplantation in allogeneic settings has been proposed, and tissue transplantation derived from this banking process has just commenced. The authors of this observational study analyzed 5,017 pairs of single cord blood transplantations with data on HLA-A, B, C, and DRB1 allele typing, identifying 39 pairs of HLA homozygous donors to HLA heterozygous patients. Notably, all 39 HLA homo to hetero pairs successfully engrafted neutrophils, with the exception of one pair that experienced an early death. Furthermore, all 30 evaluable pairs engrafted platelets. Acute graft-versus-host disease (GVHD) of grades II-IV and grades III-IV was observed in 17 and three of the 38 evaluable pairs, respectively. Competing risk regression analysis indicated a favorable likelihood of neutrophil engraftment, along with an increased risk of acute GVHD compared to HLA-matched cord blood transplants (CBTs). Among the 39 homo to hetero pairs, 37 maintained conserved extended HLA haplotypes specific to certain ethnicities. These findings support their preliminary results from six HLA homo CBTs and reveal a newly observed trend towards a higher incidence of acute GVHD. Importantly, they suggest that HLA-homo iPSC transplantation may offer advantageous engraftment, highlighting the benefits of banking iPSCs with homozygous major conserved extended HLA haplotypes [50].

Scaffolds

Tissue engineering is a contemporary field focused on the replacement and regeneration of tissues. This interdisciplinary domain involves the integration of biomaterials, such as scaffolds, alongside cells and growth factors to create new tissue [51]. It also addresses challenges encountered in autologous and allogeneic tissue repair, including insufficient tissue availability, complications at the donor site, and unintended immune reactions [52]. The scaffold serves as a framework for embedding cells and growth factors, mimicking the extracellular matrix to support and restore tissue function. Key properties that must be taken into account when designing the scaffold include high porosity, pore interconnectivity, biocompatibility, biodegradability, and mechanical strength [53].

In a recent observational study [54], a total of 155 patients with symptomatic partial meniscal defects were implanted with a polyurethane scaffold in a prospective, single-arm, multicentric study. The endpoints included scaffold removal, conversion to a meniscal transplant, and unicompartmental or TKA. Eighteen patients (11.6%) were lost to follow-up. The patients who remained in the study showed significant clinical improvement post-surgery, as indicated by various outcome measures. MRI scans of 56 patients revealed that the implants were generally smaller in size compared to the native meniscus and exhibited an irregular surface at the five-year follow-up. During the follow-up period, 87.6% of the implants survived. Specifically, at the five-year mark, 87.9% of the medial scaffolds and 86.9% of the lateral scaffolds were still functioning. The polyurethane meniscal implant effectively improved knee joint function and reduced pain in patients with segmental meniscal deficiency over the five-year period post-implantation. The MRI appearance of the scaffold differed from that of the original meniscal tissue at the midterm follow-up. The survival rates of 87.9% for the medial scaffolds and 86.9% for the lateral scaffolds in this study compare favorably with published rates for meniscal allograft transplantation following total meniscectomy [54]. Moreover, a case series assessed the safety and efficacy of a 3D-printed scaffold in chronic wound treatment. This scaffold is composed of both natural and synthetic materials and can be manufactured in either powder or membrane form. In this study, patients with pressure ulcers (PUs) and/or diabetic foot ulcers (DFUs) were recruited for the study. We employed two approaches: the use of 3D-printed scaffolds on their own and a combination of 3D-printing powder mixed with platelet-rich fibrinogen (PRF). In the case of the patient treated with the 3D-printed scaffold membrane (n=1), their PU healed in 28 days. For the two patients treated with the 3D-printed scaffold powder (n=2), their PUs healed in 54 days. Among the patients receiving the 3D-printed powder combined with PRF, one patient with a PU healed in 11 days, while the patient with a DFU healed in 14 days. All clinicians found the 3D-printed scaffold to be 'easy' or 'very easy' to use, and patients reported their comfort during wear and at dressing changes as 'good' or 'very good'. This study showed that the 3D-printed scaffold is easy to use, has the potential to enhance wound healing rates, and offers a safe and effective method for treating chronic wounds [55].

3D Printed

The use of 3D printing in healthcare has grown significantly because it can create customized medical devices tailored to individual patients. In fields such as plastic surgery and prosthetics, this technology provides precise solutions that meet specific needs. By employing 3D scanning methods along with CAD software, accurate models are produced and converted into physical items through 3D printing [56].

A recent study that combined 3D-printed techniques with some SC therapies in plastic surgery investigated whether a 3D-printed beta-tricalcium phosphate (β-TCP) scaffold, combined with growth factors, fibrin glue, and autologous bone marrow-derived mesenchymal SCs, could facilitate bone regeneration and new bone formation. Twenty β-TCP scaffolds were used, with 10 containing osteogenic mesenchymal SCs and fibrin glue (group A) and 10 serving as controls with only β-TCP and fibrin glue (group B). The scaffolds were assessed for cell infiltration, migration, and proliferation under static and dynamic conditions, and evaluated for bone-like tissue formation at two, four, and eight weeks. The results demonstrated that the β-TCP scaffold combined with mesenchymal SCs significantly enhanced maxillofacial skeletal repair, presenting a promising alternative to autologous bone grafts and various other regenerative modalities [57].

Challenges and Future Directions

While SC therapy in plastic surgery holds great promise, there are challenges such as ensuring the safety, efficacy, and ethical considerations of SC use. Ongoing research and clinical trials are crucial to overcoming these hurdles and expanding the applications of SC therapy in plastic surgery.

SC therapy faces significant hurdles in ensuring both safety and ethical considerations. The potential for tumor formation, immune system rejection, and unanticipated side effects require extensive preclinical and clinical testing. Moreover, the ethical sourcing of SCs, especially embryonic SCs, remains a point of ongoing discussion [58]. Standardizing protocols for SC isolation, culture, and application is essential for ensuring consistency and reliability in research outcomes. Variability in these processes can result in inconsistent results, which may hinder the translation of findings into clinical practice. Additionally, regulatory frameworks need to evolve alongside technological advancements to ensure that SC therapies are both safe and effective for patients [59]. Sourcing sufficient quantities of viable SCs remains a challenge. While ADSCs and BMSCs are commonly used, their proliferation and differentiation capabilities can vary. Ensuring a reliable and abundant supply of high-quality SCs is essential for successful therapeutic outcomes [60]. One of the major obstacles to the widespread adoption of SC therapies is the high cost associated with these treatments. To make these therapies more accessible to a larger patient population, it is crucial to develop cost-effective methods for cell isolation, expansion, and application. This may involve optimizing existing protocols, leveraging technological advancements, and exploring alternative sources of SCs that are more readily available and economical to harvest. By addressing the financial barriers, SC therapies can potentially reach a wider audience and have a greater impact on healthcare outcomes [61].

Advancements in genetic engineering and CRISPR (short for clustered regularly interspaced short palindromic repeats) technology offer exciting potential for developing more effective and safer SC therapies. These innovative techniques can improve the regenerative capabilities of SCs while minimizing the risk of adverse effects. By enabling precise modifications at the genetic level, researchers can optimize SC functions and tailor therapies to better meet patient needs, ultimately enhancing treatment outcomes [62]. The creation of innovative biomaterials and scaffolds can enhance the delivery of SCs and their integration into target tissues. These materials not only provide essential structural support but also foster an environment that promotes tissue regeneration. By optimizing the interaction between SCs and their surroundings, these advanced biomaterials can significantly improve the effectiveness of regenerative therapies [63]. The incorporation of advanced imaging techniques and real-time monitoring can significantly improve the precision and effectiveness of SC therapies. Technologies such as MRI and ultrasound can be utilized to monitor cell migration, differentiation, and tissue regeneration, providing valuable insights into the therapeutic process. By enabling continuous observation, these techniques can help optimize treatment outcomes and ensure that SCs are functioning as intended within the target tissues [64].

Generally, SC therapy in plastic surgery utilizes a variety of techniques, each with unique advantages and applications. These innovations are paving the way for more effective and natural-looking outcomes in reconstructive and aesthetic procedures.

## Conclusions

Our bibliographic research conducted us to report that SC therapy in plastic surgery leverages the regenerative capabilities of mesenchymal SCs, such as ADSCs and BMSCs, to enhance tissue repair and aesthetic outcomes. Methods include using ADSCs in fat grafting and facial rejuvenation, BMSCs for wound healing, and PRP to support SC effectiveness. Additionally, scaffolds and cellular reprogramming techniques are employed to guide SC growth and create customized cell populations for complex tissue engineering and regenerative procedures. In addition, and according to the data of the current review, one of the key advantages of SC therapy is its potential to promote faster healing and reduce scarring, particularly in reconstructive surgeries and wound healing. The incorporation of ADSCs, BMSCs, and PRP has shown promising results in improving skin texture and promoting tissue regeneration. Thus, despite the exciting advancements in SC therapy, there are still challenges that need to be addressed. Standardization of protocols, cost-effectiveness, and regulatory hurdles remain areas of ongoing research and development. However, the immense potential of SC therapy in plastic surgery is undeniable, and with continued progress, these challenges will be overcome. By harnessing the power of regenerative medicine, plastic surgeons can provide their patients with superior outcomes, improved quality of life, and a renewed sense of confidence.
